# Effect of family history, occupation and diet on the risk of Parkinson disease: A case-control study

**DOI:** 10.1371/journal.pone.0243612

**Published:** 2020-12-17

**Authors:** Margherita Torti, Chiara Fossati, Miriam Casali, Maria Francesca De Pandis, Paola Grassini, Fabiana Giada Radicati, Paola Stirpe, Laura Vacca, Ivo Iavicoli, Veruscka Leso, Marcello Ceppi, Marco Bruzzone, Stefano Bonassi, Fabrizio Stocchi

**Affiliations:** 1 San Raffaele Pisana Institute for Research and Medical Care, Clinical Trial Center, Rome, Italy; 2 Department of Movement, Human and Health Sciences, University of Rome “Foro Italico”, Rome, Italy; 3 San Raffaele Cassino, Cassino, Italy; 4 Department of Public Health University of Naples Federico II, Naples, Italy; 5 Unit of Clinical Epidemiology, Ospedale Policlinico San Martino, Genoa, Italy; 6 Unit of Clinical and Molecular Epidemiology, Institute for Research and Medical Care, San Raffaele Pisana, Rome, Italy; 7 Department of Human Sciences and Quality of Life Promotion, San Raffaele University, Rome, Italy; IRCCS Istituto Delle Scienze Neurologiche di Bologna, ITALY

## Abstract

**Background:**

The aetiology of Parkinson’s disease (PD) is still very controversial, with a peculiar lack of established risk factors or protective behavior.

**Methods:**

We carried out a case–control study of 634 idiopathic PD patients admitted from 2011 to 2015 to two hospitals located in central Italy and 532 controls matched by hospital, gender and age (± 5 years). The study questionnaire included questions on host factors, family history, residence, occupation and lifestyle. Odds ratios (ORs) for PD and 95% confidence intervals (CIs) were estimated with logistic regression, adjusting for actual and potential confounders.

**Results:**

A lower OR was observed in females (0.74; 95%CI:0.58–0.96), while older age classes showed a constantly increased risk for PD (p<0.005) starting from the class 65–69 years. Subjects who reported a first degree relative affected by PD showed a borderline increase which was more evident in those enrolled in the urban center of Rome (OR = 1.65; 95%CI: 1.09–2.50). Significant reduction of the risk was associated to current smoking (OR = 0.48; 95%CI: 0.24–0.54), and to vegetables consumption (p<0.03), while borderline increases were associated to meat and cold cut consumption. Occupational activities classified according to ISCO-08 categories did not show increased risk, while higher ORs’ were found for pilots and physicians.

**Conclusions:**

The results from this study confirmed the higher risk of PD in males and in elderly, and the inverse association with smoking habit. The possible etiological role of familial clustering, dietary habit, and some job tasks is suggested.

## Introduction

Parkinson disease (PD) is a neurodegenerative disorder that affects almost 6.3 million people worldwide, 1.2 million only in Europe [1]. Its pathogenesis is complex and is most likely determined by the interaction between the genetic background and environmental risk factors [2]. With the exception of few families with manifest Mendelian inheritance, preliminary evidence demonstrated a limited effect of genetic susceptibility in the etiology of PD, that needs to be deeply elucidated [2]. The relative risk (RR) of developing the diseasein the first-degree relatives of parkinsonian patients is higher than in the general population, in particular in case of early onset PD. The great variability that emerges from various studies is probably due to the mixed effect of genetic susceptibility and response to predisposing factors [3]. For this reason, the role of environmental and occupational risk factors has been extensively investigated in many epidemiological studies. An increased risk of PD has been associated in various studies with rural lifestyle, farming, pesticide exposure and also in populations with habitual residence in rural areas [4–9]. Environmental and occupational exposure to industrial chemicals, such as solvents and organo-halogenated compounds and metals, has also been related to PD [10–12].

As far as occupational risk factors are concerned, some occupations such as agricultural work [13, 14], teaching [15] and health care [13, 15] have been associated to an increased risk of PD, in addition to the above-mentioned exposure to heavy metals [16, 17], solvents [18] and pesticides [19–21]. However, definite conclusions cannot be drawn in this regard, considering also the lack of substantial support to the hypothesis that workplace factors may increase the risk of PD [22, 23]. Several other non-genetic risk factors have been investigated, mostly dealing with diet, including the oxidant/antioxidant effect of some food [24, 25].

While tobacco smoking has been inversely and consistently associated with the risk of PD in several studies [5], studies on other factors, including head injury or infectious disease, yielded inconsistent results [12].

Therefore, at present the etiology of PD is still very controversial. While the limited understanding of genetic and genomic susceptibility is common to most sporadic chronic diseases, the lack of established risk factors is quite peculiar of neurodegenerative diseases. The priority in the field of PD in order to develop successful strategies of prevention is to increase our knowledge concerning risk factor or protective behavior, to be applied in public health programs. These achievements must necessarily pass through large and comprehensive population-based case-control studies.

On these premises a large *vis-à-vis* case-control study has been conducted to investigate the role of an extensive battery of occupational, environmental, or lifestyle risk factors on the risk of PD.

## Materials and methods

### Study population

A study group of 548 consecutive PD patients was enrolled from the Movement Disorders outpatient clinics of the IRCCS San Raffaele Pisana hospital, located in Rome, and a second group of 86 (13.6%) PD patients was enrolled from the San Raffaele Cassino hospital located in south of Rome in an industrial area with a vast rural hinterland. A total of 634 PD cases was included in the study. All subjects were diagnosed with idiopathic PD according to UK PDS Brain Bank Criteria [26] and were *consecutively* enrolled between December 2011 and December 2015.

Newly diagnosed subjects and patients referred for the first time to both centers were considered for inclusion after confirming the diagnosis of idiopathic PD. Subjects with suspect of secondary parkinsonism or patients not able to understand and answer the study questionnaire were excluded from enrollment. All subjects with a medical history of major psychiatric disorders, and subjects unable to understand the Italian language were excluded from the study. Controls were selected from subjects referred to the different outpatient clinics of Rome and Cassino hospitals for diagnosis other than neurodegenerative disease. Main attending clinics were the cardiological, endocrinological, orthopedic, radiological and dermatological departments. Controls were matched to PD cases by reference center and were not family members of cases. A total number of 532 controls were included in the study, 446 (83.8%) from the San Raffaele center in Rome, and 86 (16.2%) from the San Raffaele Hospital in Cassino).

Subjects that accepted to participate to the research and signed the informed consent were enrolled and administered the study questionnaire in the period between 2011 and 2015. Subjects who declined to participate (approximately 15%) did it mainly for privacy reason and lack of time. Sensitive analyses did not reveal any critical differences in demographic or clinical characteristics between subjects who refused and those that accepted to participate to the study. The protocol was designed and carried out according to the Declaration of Helsinki and approved by the IRCCS San Raffaele Pisana Ethics Committee (Prot. 12/11; 2 May 2011).

### Questionnaire

All enrolled subjects were administered a questionnaire by trained personnel (MD’s and research nurses). The questionnaire included information on residence history (with complete address, periods of residence and indication of rural/urban area), education (years) and occupational histories, focusing on the job tasks held during the whole of working life. All case and control subjects, according to the longest held occupations, were grouped according to the International Standard Classification of Occupations 2008 (ISCO-08) [27] in: science and engineering professionals; health professionals; teaching professionals; business and administration professionals; information and communications technology professionals; legal, social and cultural professionals; clerical support workers; services and sales workers; skilled agricultural workers; craft and related trade workers; plant and machine operators and assemblers; armed forced occupations. Housewives and subjects whose occupational data were missing were considered as separate categories. The questionnaire included also questions on the potential exposure to toxicants during leisure time activities; current and/or past substance abuse; smoking history (current/former/never; number of cigarettes; duration, years since cessation); passive smoking history (cohabiting or working with smokers, specifying the number of hours per day plus number of days per week); family history of PD/other neurodegenerative diseases (relatives/parents/siblings) together with height, weight (current and habitual) and use of diet supplements. Dietary habit prior to PD onset was assessed with a previously validated food frequency questionnaire [28] and included type and frequency of most common foods; coffee/tea/carbonated beverages drinking (frequency; amount) as well as alcohol drinking (frequency, beverage).

In addition to the general questionnaire, for selected job categories extrapolated through the preliminary analysis, a specific exposure assessment tool was developed by I.I. and V.L. to collect additional information on the type of work activities, specific job tasks performed, workplace aspects and perceived occupational risks. The Microsoft Access database management system was used to store data and as a graphical interface for data-entry.

### Statistical methods

Patients and controls characteristics were explored using descriptive statistics, and compared with univariate analysis, using student’s *t* test, and χ^2^ test for continuous and categorical variables, respectively. The adjusted comparison between patients with Parkinson's disease and controls was carried out through the unconditional logistic regression model [29]. Confounding effect was taken into account including in the logistic model an appropriate set of actual and potential confounders related to individual characteristics, lifestyle and dietary patterns. For each model, the occurrence of over-dispersion was checked by comparing the residual deviance with its degrees of freedom. The Likelihood Ratio Test (LRT) was applied to assess the significance of each variable in the logistic model. STATA software was used for all statistical analyses [30].

## Results

The main characteristics of cases and control subjects are summarized in Table 1. Mean disease duration of cases enrolled was 7.4 ± 6.16 years (median 5.95; range: 0.005–47.07). As expected, the frequency of males was higher among cases (57.6% vs 49.1%; p<0.05). Cases were more likely to be older, never smoker, and with a relative affected by PD. The distribution of cases and controls by education, residence, and occupation classified according the ISCO-08 did not apparently show major differences. However, sub-group analysis detected that physicians and airplane pilots showed a higher proportion of cases. Clerical workers were underrepresented within cases (25.7% vs 32.9%).

**Table 1:** Selected demographic and life-style characteristics of the study population by PD status (*n (%)*).

Characteristic	PD Cases (n = 634)	Controls (n = 532)	P-value
			Fisher’s exact test
Sex			0.004
*Males*	365 (57.6)	261 (49.1)	
*Females*	269 (42.4)	271 (50.9)	
Age-class			0.001
*≤ 59 y*.	112 (17.7)	141 (26.5)	
*60–64 y*.	89 (14.0)	88 (16.5)	
*65–69 y*.	126 (19.9)	102 (19.2)	
*70–74 y*.	161 (25.4)	102 (19.2)	
*≥ 75 y*.	146 (23.0)	99 (18.6)	
Education			0.055
*Primary school (5 y*.*)*	126 (19.9)	80 (15.0)	
*Secondary school(6–8 y*.*)*	151 (23.8)	150 (28.2)	
*High school(9–14 y*.*)*	214 (33.8)	194 (36.5)	
*Academic qualification (≥15y*.*)*	140 (22.1)	103 (19.4)	
Residence			0.153
*Urban*	471 (74.3)	370 (69.5)	
*Mixed*	119 (18.8)	124 (23.3)	
*Rural*	36 (5.7)	30 (5.6)	
Smoking habit			<0.001
*Non smoker*	341 (53.8)	244 (45.9)	
*Former smoker*	228 (36.0)	187 (35.1)	
*Current smoker*	65 (10.2)	101 (19.0)	
Smoking duration			<0.001
*0*	341 (53.8)	244 (45.9)	
*1–20*	126 (19.9)	75 (14.1)	
*21–40*	105 (16.6)	118 (22.2)	
*>40*	53 (8.4)	88 (16.5)	
Pack-years			<0.001
*0*	341 (53.8)	244 (45.9)	
*1–10*	120 (18.9)	70 (13.2)	
*11–30*	97 (15.3)	109 (20.5)	
*>30*	67 (10.6)	101 (19.0)	
Passive smoking			0.292
*No*	167 (26.3)	154 (28.9)	
*Yes*	465 (73.3)	372 (69.9)	
Occupational activity			0.413
*Science and engineering professionals*	29 (4.7)	18 (3.4)	
*Health professionals*	26 (4.2)	17 (3.2)	
*Teaching professionals*	44 (7.1)	38 (7.2)	
*Business and administration professionals*	11 (1.8)	10 (1.9)	
*Information and communications technology professionals*	3 (0.5)	3 (0.6)	
*Legal*, *social and cultural professionals*	25 (4.0)	16 (3.0)	
*Clerical support workers*	160 (25.7)	173 (32.9)	
*S**ervices and sales workers*	107 (17.2)	93 (17.7)	
*Skilled agricultural workers*	11 (1.8)	9 (1.7)	
*Craft and related trade workers*	65 (10.4)	43 (8.2)	
*Plant and machine operators and assemblers*	64 (10.3)	45 (8.6)	
*Armed forced occupations*	11 (1.8)	4 (0.8)	
*Housewives*	65 (10.4)	57 (10.8)	
*Prisoners*	1 (0.2)	0 (0.0)	
*Physicians*	15 (2.4)	5 (0.9)	
*Pilots*	6 (0.9)	1 (0.2)	
Family history of PD *(all relatives/1*^*st*^ *degree relatives)*			<0.001/ 0.279
*No*	490 (77.3)/ 551 (86.9)	467 (87.8)/ 474 (89.1)	
*Yes*	144 (22.7)/ 83 (13.1)	65 (12.2)/ 58 (10.9)	

The distribution of study groups by consumption of selected food items is reported in Table 2. No differences in both, the mean customary and current BMI values, were observed in the cases and in the control group. PD cases showed a lower consumption of vegetables (p<0.05), coffee (p = 0.06) and a higher consumption of meat (p<0.05), cold cuts (p = 0.014) and carbonated drinks (p = 0.043).

**Table 2:** Dietary profile of the study population by PD status (*values are mean ± SD or n (%)*).

Characteristic	PD Cases (n = 634)	Controls (n = 532)	P-value
			Fisher’s exact test
BMI			
*Customary*	25.8*±*3.9	25.8*±*4.1	0.841*
*Current*	26.0*±*4.2	26.5*±*4.3	0.071*
Fruit consumption			0.351
*Never*	1 (0.2)	4 (0.8)	
*Once a month*	6 (0.9)	6 (1.1)	
*Once a week*	93 (14.7)	67 (12.6)	
*Once a day*	532 (83.9)	447 (84.0)	
Vegetable consumption			0.042
*Never*	3 (0.5)	1 (0.2)	
*Once a month*	9 (1.4)	6 (1.1)	
*Once a week*	186 (29.3)	119 (22.4)	
*Once a day*	434 (68.5)	397 (74.6)	
Meat consumption			0.040
*Never*	26 (4.1)	28 (5.3)	
*Once a month*	63 (9.9)	63 (11.8)	
*Once a week*	486 (76.7)	407 (76.5)	
*Once a day*	56 (8.8)	26 (4.9)	
Fish consumption			0.549
*Never*	19 (3.0)	12 (2.3)	
*Once a month*	134 (21.1)	100 (18.8)	
*Once a week*	468 (73.8)	400 (75.2)	
*Once a day*	10 (1.6)	12 (2.3)	
Cold cuts consumption			0.014
*Never*	41 (6.5)	40 (7.5)	
*Once a month*	117 (18.5)	113 (21.2)	
*Once a week*	394 (62.1)	334 (62.8)	
*Once a day*	79 (12.5)	37 (7.0)	
Coffee consumption			0.061
*Never*	71 (11.2)	48 (9.0)	
*Once a month*	21 (3.3)	12 (2.3)	
*Once a week*	22 (3.5)	8 (1.5)	
*Once a day*	518 (81.7)	456 (85.7)	
Coffee consumption *(cups/Day)*			0.120
*0–1*	259 (40.9)	187 (35.2)	
*2*	207 (32.6)	174 (32.7)	
*≥3*	166 (26.2)	162 (30.4)	
*Missing*	2 (0.3)	9 (1.7)	
Tea consumption			0.361
*Never*	257 (40.5)	239 (44.9)	
*Once a month*	168 (26.5)	130 (24.4)	
*Once a week*	86 (13.6)	69 (13.0)	
*Once a day*	121 (19.1)	86 (16.2)	
Cola consumption			0.043
*Never*	321 (50.6)	297 (55.8)	
*Once a month*	189 (29.8)	156 (29.3)	
*Once a week*	77 (12.1)	50 (9.4)	
*Once a day*	44 (6.9)	21 (3.9)	
Wine consumption			0.147
*Never*	138 (21.8)	141 (26.5)	
*Occasionally*	242 (38.2)	194 (36.5)	
*Yes*	253 (39.9)	194 (36.5)	
Beer consumption			0.381
*Never*	253 (39.9)	232 (43.6)	
*Occasionally*	352 (55.5)	277 (52.1)	
*Yes*	27 (4.3)	19 (3.6)	

*t-test.

The results of logistic regression analysis are reported in Tables 3–5. As regards to demographic parameters, a significantly lower odds ratio (OR) was observed in females (0.74; 95% CI:0.58–0.96), while older age classes showed a constantly increased risk (p = 0.005), with statistical difference from the reference value (≤ 59 years) starting from the class 65–69 years. Subjects with higher education have generally lower, non-significant risk, and the same is observed for those living in a mixed or rural environment, i.e., 0.72 (95%CI: 0.53–0.98), and 0.97 (95%CI: 0.57–1.65) respectively. A specific item in the questionnaire investigated the presence of relatives affected by PD with a distinct question restricted to first degree relatives. Compared to those with negative family history of PD, those who reported an affected relative had a highly significant risk, OR = 2.22; 95% CI: 1.59–3.10. When the interview was restricted to first degree relatives the risk decreased to a borderline significant OR of 1.34 (95%CI: 0.92–1.93). Given the heterogeneous social background of the two clinical centers, sensitivity analyses were performed to evaluate risks by center. A significantly higher OR for first degree familiarity was found in the center located in Rome (1.65; 95%CI: 1.09–2.50).

**Table 3:** Association of demographic parameters with PD status (OR and 95% CIs*).

	Odds Ratio	95% Confidence Interval	P value (LRT)
Sex			0.021
*Males*	Ref.	-	
*Females*	0.74	0.58–0.96	
Age-class			0.005
*≤ 59 y*.	Ref.	-	
*60–64 y*.	1.28	0.86–1.92	
*65–69 y*.	1.57	1.08–2.30	
*70–74 y*.	1.92	1.33–2.79	
*≥ 75 y*.	1.76	1.21–2.57	
Education			0.376
*Primary school (5 y*.*)*	Ref.	-	
*Secondary school(6–8 y*.*)*	0.75	0.51–1.10	
*High school(9–14 y*.*)*	0.87	0.60–1.26	
*Academic qualification ≥15y*.*)*	0.98	0.65–1.48	
Residence			0.103
*Urban*	Ref.	-	
*Mixed*	0.72	0.53–0.98	
*Rural*	0.97	0.57–1.65	
Family history of PD			<0.001
*(all relatives)*			
*No*	Ref.	-	
*Yes*	2.22	1.59–3.10	
Family history of PD			0.124
*(1*^*st*^ *degree relatives)*			
*No*	Ref.		
*Yes*	1.34	0.92–1.93	

*ORs and 95% CIs computed from an unconditional logistic regression model and adjusted for sex, age, smoking habit, intake of vegetable, meat, and cold cuts.

**Table 4:** Association of selected food items with PD status (OR and 95% CIs*).

	Odds Ratio	95% Confidence Interval	P value	P value for trend
			(LRT)	
Meat consumption			0.085	0.028
*Never*	Ref.	-		
*Once a month*	0.93	0.48–1.80		
*Once a week*	1.23	0.69–2.19		
*Once a day*	1.99	0.95–4.17		
Cold cuts consumption			0.066	0.041
*Never*	Ref.	-		
*Once a month*	0.93	0.55–1.58		
*Once a week*	1.09	0.67–1.77		
*Once a day*	1.78	0.97–3.30		
Vegetable consumption			0.033	0.005
*Never*	Ref.	-		
*Once a month*	0.50	0.04–6.48		
*Once a week*	0.55	0.05–5.53		
*Once a day*	0.37	0.04–3.67		
Coffee consumption			0.178	0.169
*Never*	Ref.	-		
*Once a month*	1.08	0.47–2.45		
*Once a week*	1.76	0.70–4.39		
*Once a day*	0.80	0.53–1.21		
Carbonated drinks consumption			0.058	0.007
*Never*	Ref.	-		
*Once a month*	1.18	0.89–1.56		
*Once a week*	1.40	0.93–2.12		
*Once a day*	1.95	1.11–3.44		
Wine consumption			0.495	0.240
*Never*	Ref.	-		
*Occasionally*	1.22	0.88–1.68		
*Yes*	1.12	0.80–1.58		
Beer consumption			0.452	0.229
*Never*	Ref.	-		
*Occasionally*	1.17	0.91–1.52		
*Yes*	1.22	0.64–2.35		

*ORs and 95% CIs computed from an unconditional logistic regression model and adjusted for sex, age, smoking habit, intake of vegetable, meat, and cold cuts.

**Table 5:** Association of selected sectors of occupation with PD status (OR and 95% CIs*).

Occupational activity	PD Cases (n = 634)	Controls (n = 532)	Odds Ratio	95% Confidence Interval	P value (LRT)
					0.893
*Clerical support worker*	160	173	Ref.	-	
*Science and engineering professionals*	29	18	1.34	0.69–2.61	
*Health professionals*	26	17	1.59	0.81–3.12	
*Teaching professionals*	44	38	1.33	0.79–2.23	
*Business and administration professionals*	11	10	0.85	0.34–2.14	
*Information and communications technology professionals*	3	3	1.36	0.26–7.01	
*Legal*, *social and cultural professionals*	25	16	1.33	0.67–2.64	
*S**ervices and sales workers*	107	93	1.21	0.83–1.76	
*Skilled agricultural workers*	11	9	1.04	0.41–2.67	
*Craft and related trade workers*	65	43	1.29	0.81–2.06	
*Plant and machine operators and assemblers*	64	45	1.37	0.86–2.18	
*Armed forced occupations*	11	4	2.43	0.73–8.13	
*Housewives*	65	57	1.25	0.78–2.00	
*Physicians*	15	5	3.07	1.05–9.01	
*Pilots*	6	1	4.73	0.54–41.7	

*ORs and 95% CIs computed from an unconditional logistic regression model and adjusted for sex, age, smoking habit, intake of vegetable, meat, and cold cuts.

An extensive description of the association between PD risk and various features of smoking habit is reported in S1 Table. A strong reduction of risk can be observed in current smokers, who showed a 52% lower PD risk (OR = 0.48; 95%CI: 0.33–0.69). The observed protective effect of smoking was significantly modified by the years since quitting for former smokers, although the diminishing protective effect after cessation of smoking was not homogeneous. Consistently, also parameters of smoking duration (p<0.001) and intensity (p<0.001) showed inverse trends with PD risk. No association was found with passive smoking.

The association of PD risk with the frequency of consumption of selected food items is reported in Table 4. An increasing trend can be observed with the consumption of meat. The overall LRT is not significant (p = 0.085), but the trend is significant, and those eating meat everyday showed a doubled OR when compared to those who never eat meat (OR = 1.99; 95% CI 0.95–4.17). An increased risk of PD is also reported in those drinking carbonated drinks daily (OR = 1.95; 95% CI 1.11–3.44), or even weekly (OR = 1.40; 95% CI 0.93–2.12). Cold cuts consumption exhibits an increasing trend in PD risk (p = 0.041). A protective effect has been associated to vegetable consumption (LRT p = 0.033), and to a non-significant extent to daily coffee consumption (OR = 0.80; 95% CI 0.53–1.21). No association with PD risk was found for other food items. Eventually, the risk of PD was associated to the occupational sector, as coded by ISCO-08. Using clerical support workers as referent, several sectors showed higher risks though not significant. In particular, the health professionals, with an OR of 1.59 (95% CI 0.81–3.12), plant and machine operators and assemblers (1.37; 95% CI 0.86–2.18), craft and related trade workers (1.29; 95% CI 0.81–2.06), services and sales workers (1.21; 95% CI 0.83–1.76), armed forces occupations (2.43; 95% CI 0.86–8.18). Additional analyses were performed in subgroups of these main sectors extrapolated according to the performed tasks, showing interesting results for some specific categories such as pilots (OR = 4.73; 95% CI 0.54–41.7), or reaching a significant evidence for physicians (OR = 3.07; 95% CI 1.05–9.01). Specific occupational features of these latter occupations were specifically investigated. In the group of medical doctors, neither working in laboratory or in surgery rooms, nor the distinction between working in outpatient or inpatients activities resulted associated to the risk of PD. In addition to these results, nor biological, physical, or chemical occupational risks reported by these subjects could be associated to disease onset. Similarly, logistic features, such as shift, night or overtime works did not result to influence the risk of PD in this occupational setting. No significant risk differences emerged between civil or military aircraft pilots, as well as short, medium or long-haul pilots compared to controls, and also chemical, physical, and organizational factors failed to be significantly associated with PD onset.

## Discussion and conclusion

The results from the present case-control study confirmed the etiological role of some factors already described in several studies, such as the higher risk of PD for males and for older age-classes, and the inverse association with smoking habit. On the other hand, the availability of a comprehensive questionnaire which included host factors, residence, occupation, and several features of lifestyle, allowed to study a number of potential risk factors insufficiently investigated so far. This approach suggested that a family history of PD, the consumption of meat, cold cuts and carbonated drinks, working as a medical doctor or as a pilot increased the risk of PD, while a constant consumption of vegetables reduced the risk, especially in those eating vegetables daily. The negative association with vegetable consumption were quantitatively unstable due to the small number of subjects in the reference category of those who never have vegetables. A more balanced comparison of subjects who eat vegetables everyday vs those who have vegetables once per week confirmed the trend reported in Table 5 (OR = 0.69, p<0.01).

The most convincing evidence from our study concerned the inverse association with smoking habit [31, 32]. In agreement with existing literature the protective effect of smoking was higher in those subjects with a long history of smoking (OR = 0.36; 95% CI 0.24–0.54 in those smoking 40 years or more) and in those smoking more than 30 pack-years (OR = 0.40; 95% CI 0.27–0.58), while no consistent results were found in the group of former smokers with the years since cessation. Altogether, these results, in association with those referring to the effect of gender and age confirm the high degree of internal validity of this study, an important condition that increases the credibility of innovative findings referring to other possible risk factors. As concerns other voluptuary habits, i.e. the impact of alcohol assumption on PD risk, no consistent pattern was found in our data, reflecting the extreme variability of results from the literature [24, 33], characterized by the heterogeneity of methods used to assess alcohol consumption, the genetic variability of population settings, and the contrasting inclusion criteria.

The presence of an inherited predisposition to PD has been the object of several investigations, as extensively reported by Thacker and Ascherio [3]. As regards our results we clearly showed that the use of non-first-degree-only relatives results in a biased approached, due to tendency of individuals with PD to be more likely aware of PD diagnosis in higher-order relatives than controls, i.e., OR = 2.22 vs 1.34, respectively. On the other hand, our approach, based on a *vis-à-vis* contact, a reconstruction of the family composition (number of children), and an estimate of OR for subjects with positive family history adjusted for a large battery of predictive factors, supports the conclusion that the increased probability of PD in subjects positive for familial history of PD may have been overestimated. The adjusted estimation procedure yields in a level of risk which is in the same range of other major multifactorial diseases, such as cancer [34] and cardiovascular diseases [35]. The confirmation of how several factors may change the strength of the association between positive familial history of PD and individual risk comes from the difference between the two clinical centres contributing cases and controls to this study. The higher and significant risk found in subjects from the centre in Rome (OR = 1.65; 95%CI: 1.09–2.50), given the standard methods used in the assessment, supports the hypothesis that a shared environment or a population setting may modify the level of inherited susceptibility.

As regards the different components of the shared environment there is compelling evidence that a proper diet can promote healthy aging and prevent certain environmental diseases, although whether this would be the case in PD is still controversial. Previous studies have presented inconsistent results concerning the association between PD risk and the consumption of different meats and vegetables [24]. In our study PD cases showed a lower consumption of vegetables and a higher consumption of meat and cold cuts; the protective effect of caffeine has been confirmed [36], although the high coffee consumption in Italy (83.6% of the whole study group had one or more cups per day) has most likely reduced the strength of the association. Interestingly these results overlap those of other major multifactorial diseases, including cancer, cardiovascular and other inflammatory diseases [34, 35], supporting the view that the benefits of a healthy dietary pattern reflect the synergies and cumulative effects of different nutrients, rather than being the result of a single nutrient.

The effect of work on the risk of PD is relatively understudied and poorly understood. Most previous studies of occupation and PD have focused on exposures to toxicants (e.g., pesticides, solvents, lead, welding fume, and electromagnetic fields) or low socioeconomic status occupations, such as farming, construction, production, and military service [12, 13, 18]. More recently higher socio-economic status occupations have been investigated (e.g., computer and mathematical; architecture and engineering; legal; and education, training, and library occupations) [37], although general belief is that the role of occupation in the aetiology of PD is minimal. The general results of our study are in agreement with this conclusion, as we can find in a number of occupational activities such as health professionals, plant and machine operators/assemblers, craft and related trade workers, services and sales workers as well as armed forced employees, only a borderline increased risk for PD. On the other hand, subgroup analysis demonstrated that specific job titles may be associated with PD, such as in the case of pilots and physicians. Actually, the increased risk for PD in the medical profession (OR = 3.07; 95% CI 1.05–9.01 in our study) has been previously described in literature [13, 15] and it has been usually associated with a greater exposure of this category to infectious agents, supporting the “neuro-inflammatory” hypothesis in the PD pathogenesis [38, 39]. Despite the careful exposure assessment performed in our study (all 20 medical doctors were re-interviewed using an *ad hoc* questionnaire on their work) no definite conclusions can be currently extrapolated concerning possible occupational factors, in terms of specific tasks performed, perceived workplace risks as well as logistic factors, influencing such association. This lack of evidence may in part be due to the limited number of investigated subjects in each subgroup and requires that more detailed occupational investigation should be performed based on specific job task-exposure assessment and the interpretation of underlined mechanisms of action. The possible exposure to biological and chemical agents, ionizing or non-ionizing radiations, shift-works, sedentary works or workload activities as well as job strain may act as triggers for chronic mild inflammatory status in the *substantia nigra*, which makes dopaminergic neurons vulnerable to degeneration. All these aspects should be carefully verified in future *ad hoc* multi-centric, epidemiological investigations based on larger homogeneous groups of workers. An interesting hypothesis suggests that the early deterioration of dopaminergic pathways producing subtle, sub-clinical effects long before the onset of symptoms, may induce character changes addressing future patients towards working careers with particular features, i.e. less physical fatigue, high demand for precision [40].

The present study has several strengths, including the prospective collection of cases, the large sample size, which makes this study among the largest with direct interview to cases and controls, the comprehensive questionnaire covering host factors, life-style, diet and occupation, which allowed to estimate adjusted OR’s from regression models with the best goodness-of-fit. Obviously, the study has also a number of limitations. The presence of recall bias by subjects affected by PD, for those variables that may have been associated to the disease, i.e. familiarity and occupation, was addressed restricting to first degree relatives the familial relationships and performing a dedicated exposure assessment for those occupations which resulted associated to PD. As regards dietary habit, due to the limited evidence available, there is not a common believe that diet may be associated to the risk of developing PD, and therefore the presence of recall bias it is quite unlikely. On the other hands, since the questionnaire investigated dietary habits prior to PD diagnosis, a long-term recall bias cannot be ruled out. The study was performed in two different clinical centers. Despite the common geographical location in the center of Italy, the two centers differed for the socioeconomic status of the two populations, a condition which may have determined an incomplete adjustment for confounding. A general limitation of the study is also the lack of specific analyses concerning Parkinson’s disease subtypes, which in some papers have been reported to have a differential sensitivity to potential risk factors [14]. Additional re-analysis of data based on clinical PD subtypes may provide a critical improvement of current findings. The presence of numerous hypotheses tested may have generated a multiple comparison issue. Due to this condition, some exposure could have been identified as significantly associated to PD just by chance, and their p value may be unreliable. A correction was not applied since all covariates associated to the risk of PD were previously known as possible risk factors or had a strong mechanistic background that supported the results.

In conclusion, this study has confirmed some of the associations already present in literature but has also highlighted an increased risk for PD in some peculiar occupational activities and an important link to diet that could open the door to new lines of research. Furthermore, from our results clearly emerges that to understand the complexity of exposures faced throughout the lifespan, emerging techniques of biomonitoring should be integrated, with the aim of identifying strategies to transition exposure research toward exposomics and to start modelling a holistic strategy of prevention, based on genomics, clinical features, personality, lifestyle, aging, and comorbidities.

## Supporting information

S1 Table Association of different indexes of smoking habit with PD status (OR and 95% CIs*). *ORs and 95% CIs computed from an unconditional logistic regression model and adjusted for sex, age, intake of vegetable, meat, and cold cuts. ** Adjusted also by active smoke.
(DOC)

S1 Dataset Dataset generated during the study. (XLSX)
