# Supplementary material for: Effect of family history, occupation and diet on the risk of Parkinson disease: A case-control study
Source: PLoS One. 2020 Dec 17;15(12):e0243612. doi: 10.1371/journal.pone.0243612 (PMC7746265; doi:10.1371/journal.pone.0243612)
Supplement: S1 Table — *ORs and 95% CIs computed from an unconditional logistic regression model and adjusted for sex, age, intake of vegetable, meat, and cold cuts. ** Adjusted also by active smoke. (DOC) [file pone.0243612.s001.doc]

**S1 Table.** Association of different indexes of smoking habit with PD status (OR and 95% CIs^*^).

|  | Odds Ratio | 95% Confidence Interval | P value (LRT) |
| --- | --- | --- | --- |
| Smoking habit |  |  | <0.001 |
| *Non smoker* | Ref. | - |  |
| *Former smoker* | 0.76 | 0.58-1.01 |  |
| *Current smoker* | 0.48 | 0.33-0.69 |  |
| Smoking duration *(years)* |  |  | <0.001 |
| *0* | Ref. | - |  |
| *1-20* | 1.09 | 0.77-1.54 |  |
| *21-40* | 0.62 | 0.45-0.86 |  |
| *>40* | 0.36 | 0.24-0.54 |  |
| Pack-years |  |  | <0.001 |
| *0* | Ref. | - |  |
| *1-10* | 1.15 | 0.81-1.63 |  |
| *11-30* | 0.58 | 0.41-0.81 |  |
| *>30* | 0.40 | 0.27-0.58 |  |
| Passive smoking** |  |  | 0.096 |
| *No* | Ref. | - |  |
| *Yes* | 1.26 | 0.96-1.65 |  |
| Years since Cessation *(only former smokers) (years)* |  |  |  |
| *Non smokers* | Ref. |  | 0.005 |
| *> 20 years* | 0.56 | 0.40-0.79 |  |
| *11-20 years* | 1.11 | 0.69-1.78 |  |
| *≤10 years* | 0.78 | 0.47-1.30 |  |

*^*^ORs and 95% CIs computed from an unconditional logistic regression model and adjusted for sex, age, intake of vegetable, meat, and cold cuts.*

*** Adjusted also by active smoke*
